# Efficacy of Combination Chemo-Immunotherapy as a First-Line Treatment for Advanced Non-Small-Cell Lung Cancer Patients With HER2 Alterations: A Case Series

**DOI:** 10.3389/fonc.2021.633522

**Published:** 2021-04-20

**Authors:** Shuang Zhao, Xinghong Xian, Panwen Tian, Weimin Li, Ke Wang, Yalun Li

**Affiliations:** ^1^ Department of Respiratory and Critical Care Medicine, West China Hospital, Sichuan University, Chengdu, China; ^2^ Clinical Medicine of West China Medical School/West China Hospital, Sichuan University, Chengdu, China

**Keywords:** human epidermal growth factor receptor2, immunotherapy, chemotherapy, non-small-cell lung cancer, prognosis

## Abstract

**Objective:**

Although the treatment of non-small-cell lung cancer (NSCLC) patients with human epidermal growth factor receptor 2 (HER2) alterations has been studied for years, the overall response rate (ORR) of these patients is still unsatisfactory, and more therapeutic strategies are needed. Little is known about the combination of chemo- and immunotherapy in HER2-altered lung cancer treatment.

**Materials and Methods:**

We report five cases of advanced NSCLC with HER2 insertion mutation or amplification treated with immunotherapy combined with chemotherapy as the first-line treatment. The HER2 alteration type, duration of treatment and survival were also analyzed.

**Results:**

The five advanced NSCLC patients, three with HER2 mutations and two with HER2 amplifications, received chemo-immunotherapy as the first-line treatment. The average patient age was 54.6 years. Three patients were females, and two were males. Among all the patients, only one had a smoking history. The immunotherapies used were as follows: two patients were treated with sintilimab, and three patients were treated with pembrolizumab. Only one patient had squamous carcinoma, and she was also the only patient with a complete response (CR). The progression-free survival (PFS) ranged from 2-12 months, with a median PFS of 8.0 months.

**Conclusions:**

Chemo-immunotherapy may be a promising first-line treatment option for NSCLC patients with HER2 alterations. Further clinical trials are required to confirm this therapeutic option.

## Introduction

Lung cancer is known as one of the deadliest cancers worldwide and causes more deaths than prostate, breast, brain and colorectal cancers combined (1). Non-small-cell lung cancer (NSCLC) comprises approximately 85% of all lung cancer cases (2). Human epidermal growth factor 2 (HER2) is a rare oncogenic driver that is altered in 1% to 3% of NSCLC patients (3). The main types of HER2 alterations in lung cancer include gene insertion mutation, gene amplification and protein overexpression (4). Chemotherapy remains an important component of treatment for HER2-altered NSCLC patients, although HER2 positive tumors are relatively insensitive to chemoradiotherapy (5, 6). Several HER2-targeted tyrosine kinase inhibitors (TKIs) and antibodies have also been tested for the treatment of these patients. However, the overall response rate (ORR) was unsatisfactory, at only 7.4% for HER2-targeted TKIs such as neratinib, lapatinib and afatinib (7). More therapeutic strategies for NSCLC patients with HER2 alterations are needed. Little is known about the combination of immunotherapy and chemotherapy in the treatment of lung cancer with HER2 alterations. Therefore, we described five advanced NSCLC cases with HER2 mutation or amplification and immunotherapy combined with chemotherapy as the first-line treatment. We hope this case series will provide new clinical therapeutic insight for this class of patients.

## Case Presentation

From January 2019 to June 2020, five patients with advanced NSCLC with HER2 alterations and chemo-immunotherapy as the first-line treatment were admitted to the Lung Cancer Center, West China Hospital, Sichuan University. High-throughput next-generation sequencing (NGS) technology was used to assess the presence and type of HER2 alterations in the biopsy specimens of all patients. The status of PD-L1 was also tested by immunohistochemistry. This retrospective study was approved by the Committee on Medical Ethics of West China Hospital, Sichuan University.

### Patients With HER2 Mutation

Three patients harbored HER2 insertion mutations.

The first patient (Case 1) was a 37-year-old Asian female who was a never-smoker and had a symptom of severe headache. She was finally diagnosed with left lung adenocarcinoma with brain, bone, hilar and mediastinal lymph node metastases (cT2N2M1c, stage IVB). She harbored a HER2 insertion mutation in exon 20 (p. A775_G776insYVMA). EGFR, ALK, ROS-1 and PD-L1 testing was performed, and none of these targets were expressed. She received chemotherapy (carboplatin and pemetrexed) and sintilimab for 6 cycles with a partial response (PR) as her best response. Then, she experienced progressive disease (PD) with new brain metastases and was treated with pyrotinib, a pan-ErbB receptor TKI, for 2 months. However, she continued to progress with multiple new brain metastases and died because of a hemorrhagic cerebral hernia.

The second patient (Case 2) was a 65-year-old Asian man with a 10-pack-year smoking history who was diagnosed with cT4N3M1c stage IVB lung adenocarcinoma by pleural effusion smear and cytological examination. He complained of cough for 4 months. The NGS panel revealed a HER2 mutation (exon 20, p. A775_G776insYVMA) without concurrent alterations or PD-L1 expression. He was treated with carboplatin and pemetrexed combined with sintilimab for 4 cycles. His best response was stable disease (SD). Then, he experienced progressive malignant pleural effusion. One month after starting anlotinib, a chest CT scan showed a reduction in pleural effusion. The patient maintained SD until his last visit.

The third patient (Case 3) was a 52-year-old Asian man who was a never-smoker and developed cough and bloody sputum for 2 months. Chest CT showed a left lower lung mass with multiple bilateral pulmonary nodules. Brain MRI and bone single photon emission CT (SPECT) were all negative. Through percutaneous lung biopsy and left supraclavicular lymph node biopsy, he was finally diagnosed with cT4N3M1c stage IVB right lung adenocarcinoma. A HER2 mutation (exon 20, p. A775_G776insYVMA) was found from his initial molecular testing, but no other gene alterations were identified. He received carboplatin and pemetrexed chemotherapy and pembrolizumab for 2 cycles. Unfortunately, he experienced rapid progression within 2 months. He was then treated with docetaxel, carboplatin and bevacizumab for 1 month. He is currently enrolled in an EGFR/HER2-targeted TKI clinical trial (DZD9008).

### Patients With HER2 Amplification

There were two patients with HER2 amplification.

The fourth patient (Case 4) was a 72-year-old Asian female never-smoker who complained of dorsalgia for 9 months. She was diagnosed with lung adenocarcinoma by percutaneous lung biopsy. CT showed metastatic mediastinal lymphadenopathy, SPECT scanning showed multiple bone metastases, and MRI showed evidence of brain metastases. The clinical stage was cT1N2M1c stage IVB. She was found to have HER2 amplification (copy number:2.6) without other gene alterations or PD-L1 expression. She was treated with carboplatin, pemetrexed, and pembrolizumab for 9 cycles. Then, she progressed with multiple new liver and bone metastases after 12 months and started on docetaxel and pembrolizumab with radiographic evidence of SD, which was sustained for 4.0 months up to the study endpoint.

The fifth patient (Case 5) was a 47-year-old Asian female never-smoker who had no symptoms. Her chest CT scan showed multiple pulmonary nodules. She was diagnosed with cT4N0M1a stage IVA lung squamous carcinoma by percutaneous lung biopsy. NGS testing of her lung biopsy specimen was performed, and it showed HER2 amplification (copy number:3.22). EGFR, ALK, ROS-1 and PD-L1 expression were all negative. She received chemotherapy (carboplatin and pemetrexed) and pembrolizumab for 4 cycles followed by pembrolizumab maintenance therapy, with a complete response (CR) as her best response. The patient was still in remission at the endpoint of this study.

### Summary of Patients

**Figure 1:**
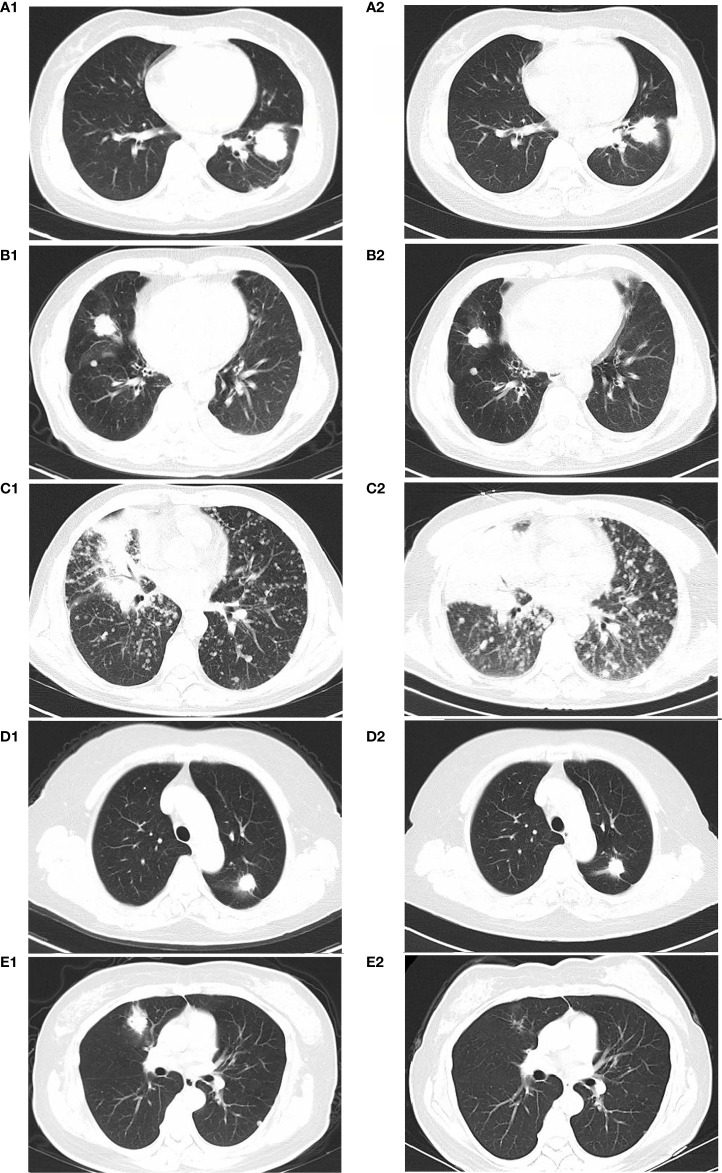
CT imaging of best disease response of patients: Case 1 at beginning of therapy **(A1)** and 4 months after receiving carboplatin+pemetrexed chemotherapy and sintilimab **(A2)** Case 2 at beginning of therapy **(B1)** and 5 months after receiving carboplatin+pemetrexed chemotherapy and sintilimab **(B2)** Case 3 at beginning of therapy **(C1)** and 2 months after receiving carboplatin+pemetrexed chemotherapy and pembrolizumab**(C2)** Case 4 at beginning of therapy **(D1)** and 10 months after receiving carboplatin+pemetrexed chemotherapy and pembrolizumab **(D2)** Case 5 at beginning of therapy **(E1)** and 6 months after treatment with carboplatin, pemetrexed, and pembrolizumab for 4 cycles followed by pembrolizumab maintenance **(E2)**.

The average patient age was 54.6 years. Three patients were females, and two were males. Among all the patients, only one had a smoking history (Case 2). The HER2 mutation type, treatments, responses and progression-free survival (PFS) for the above five patients are summarized in **Table 1**. The median PFS (mPFS) was 8 months, ranging from 2-12 months. The immunotherapies used were as follows: two patients were treated with sintilimab (Case 1 and Case 2), and three patients were treated with pembrolizumab (Case 3, Case 4 and Case 5). Only one patient (Case 5) had squamous carcinoma, and she was also the only patient with a CR. **Figure 1** displays the representative chest CT images of all the patients’ best responses.

**Table 1:** The characteristics, treatments, responses and progression free survival (PFS) for the patients.

						First-Line Treatment			Second -Line Treatment			
Patient	Histopathological type	Gender	Age	Smoking	HER2	Treatment	Best Disease Response	PFS1	Treatment	Best Disease Response	PFS2	OS
1#	ADC	female	37	Never	insertion mutation	AC+ sintilimab	PR	7m	pyrotinib	PD	4m	11m
2#	ADC	male	65	Current	insertion mutation	AC+ sintilimab	SD	8m	anlotinib	N/A	N/A	N/A >10m
3#	ADC	male	52	Never	insertion mutation	AC+ pembrolizumab	PD	2m	DC+ bevacizumab	SD	N/A	N/A >4m
4#	ADC	female	72	Never	amplification	AC+ pembrolizumab	SD	12m	D+ pembrolizumab/	SD	N/A	N/A >16m
5#	SCC	female	47	Never	amplification	AC+pembrolizumab; Pembrolizumab.	CR	N/A >12m			N/A	N/A >12m

A, pemetrexed; C, carboplatin; D, docetaxel; K, pembrolizumab; HER2, human epidermal growth factor receptor 2; ADC, adenocarcinoma; SCC, squamous carcinoma; OS, overall survival.

## Discussion

Five advanced NSCLC patients, three with HER2 mutations and two with HER2 amplifications, received chemoimmunotherapy as the first-line treatment. Among all the patients, the immunotherapies used were as follows: two patients were treated with sintilimab, and three patients were treated with pembrolizumab. Only one patient had squamous carcinoma, and she was also the only patient with a CR. The PFS ranged from 2-12 months, with a median PFS of 8.0 months.

HER2, also known as ERBB2, is a cell surface receptor tyrosine kinase of the ERBB family that is considered an oncogenic driver in many cancers, notably breast, ovarian and gastroesophageal cancers (8). The HER2 receptor is activated via heterodimerization or homodimerization with other ERBB family receptors, inducing activation of EGFR signaling (9). The main types of HER2 alterations in lung cancer include gene insertion mutation, gene amplification and protein overexpression. HER2 insertion mutations and amplifications have been reported in approximately 2-5% and 2-3% of lung adenocarcinomas, respectively (10–12).

Recently, many clinical trials have focused on HER2-targeted therapy for HER2-positive NSCLC. However, the results are ambiguous and insufficient. Trastuzumab, a monoclonal antibody for the HER2 receptor, did not show definite benefits for HER2-positive NSCLC patients (13). In contrast, TKIs targeting both HER2 and EGFR were shown to exhibit a therapeutic response. In the EUHER2 study, HER2-targeted drugs, including trastuzumab, lapatinib, neratinib and afatinib, did not show clear survival benefits compared with conventional therapy, including chemotherapy and reversible EGFR-TKIs (7). Among them, afatinib, an irreversible ERBB family blocker, might be a promising therapeutic choice for HER2-mutant NSCLC with progression after previous chemotherapy or reversible EGFR-TKI treatment (14). Afatinib showed a response rate of 18.2% and an mPFS of 3.9 months in the EUHER2 study (7), but recent phase II trials found that only patients with specific HER2 mutations had durable responses to afatinib (15). Subsequently, preliminary results for several other HER2 kinase inhibitors, including temsirolimus (16) and TAK-788 (17), have indicated an effect on regression in these patients. Thus far, HER2-targeted therapy has not achieved ideal effects, and the treatment of NSCLC patients with HER2 alterations remains a major challenge.

The advantages of HER2-targeted therapy over chemotherapy in HER2-positive NSCLC are inconclusive. Previous studies suggested that the mPFS durations of chemotherapy alone, pemetrexed ± platinum/bevacizumab, gemcitabine, taxane ± platinum/bevacizumab, and vinorelbine were 4.3 months, 6.2 months, 2.6 months, 4 months and 3.5 months, respectively. By comparison, the mPFS of HER2 TKIs was only 2.2 months (18). For HER2-mutant lung cancers, the ORR was 36%, and the mPFS was 5.1 months with chemotherapy as the first-line therapy (6). The ORR and mPFS were 50.9% and 4.8 months, respectively, with trastuzumab or ado-trastuzumab emtansine (T-DM1) (7). Therefore, chemotherapy remains an important component of treatment, while the benefit of HER2-targeted therapy is inconclusive. However, the outcome of NSCLC patients with HER2 alterations who are treated with chemotherapy can be further improved by combination treatment, and thus, additional therapies for these patients are warranted.

Immunotherapies are also worth considering for the treatment of patients with HER2 alterations. The combination treatment of pembrolizumab and chemotherapy was included in the guidelines as a first-line treatment for advanced NSCLC based on the KEYNOTE-189 trial (19, 20). Nevertheless, immunotherapy is less effective in patients with oncogenic mutations than in patients without oncogenic mutations, and anti-PD-1/PD-L1 therapy may even facilitate hyperprogression (21). Chiara Catania et al. reported a case in which nivolumab had strong antitumor activity in advanced HER2-positive lung cancer (22). Conversely, Jody C. Chuang reported that HER2-mutated NSCLC patients did not respond to nivolumab (23). Mazieres et al. reported an ORR of 7% and a median PFS of 2.5 months amongst 29 patients with HER2 altered advanced lung cancer when treated with single agent immune checkpoint inhibitors (24). In our study, five advanced NSCLC cases were described. The results showed that the PFS times of the patients ranged from 2–12 months, with an mPFS of 8.0 months with chemoimmunotherapy. Based on our experience, we propose that chemoimmunotherapy may be a hopeful first-line treatment option for NSCLC patients with HER2 alterations.

NSCLC has distinct clinical features according to the HER2 alteration type; however, both amplification and oncogenic mutation in HER2 can promote receptor hyperactivation and tumor growth (25). HER2 mutations mainly occur at exon 20 in the protein kinase domain and are recognized as primary drivers in lung cancer, similar to other oncogenic drivers, such as EGFR, ROS, ALK, KRAS and BRAF (11). In NSCLC, it is controversial whether HER2 amplification is a driver gene. Some studies have suggested that amplification of ERBB2 is a driver event specifically in oncogene-negative lung adenocarcinoma (12). However, HER2 amplification may not be associated with HER2 mutation, and they may be involved in distinct clinical entities that need different therapeutic methods (26). Case reports have suggested that pyrotinib and afatinib may also be effective for lung adenocarcinoma patients with coexisting HER2 mutation and amplification (27, 28). The anti-HER2 antibody-drug conjugates (ADCs) have shown greater clinical benefit than TKIs in HER2-amplified cancers (29), specifically T-DM1 and deruxtecan-trastuzumab (T-DXd). T-DM1 was clinically effective in ERBB2-amplified/mutant lung cancer patients, and the ORR was 51%, with a mPFS of 5 months (30, 31). For heavily pretreated HER2-mutant NSCLC, a phase I study showed that the ORR of T-DXd was 72.7%, and the median PFS was 11.3 months (95% CI, 8.1–14.3) (32). Then in an ongoing phase II study (DESTINY-Lung01 study), T-DXd demonstrated an encouraging efficacy in this molecular subset of lung cancers. The ORR was 61.9%, and the median PFS was 14.0 months (95% CI, 6.4–14.0) (33). However, all of these studies are still in phase I or II, and the sample size is relatively small. Therefore, further exploration is required to identify specific types of HER2 alterations and assess their potential as novel therapeutic targets.

There are several limitations to this study. First, the sample size was too small, and the statistical results may have biased. Therefore, more prospective, larger sample size, randomized, controlled studies are needed. Second, the PD-L1 expression of these patients was negative, so the impact of PD-L1 status on the treatment response in these patients is unknown. Finally, the follow-up time of our study was only 12 months, and the overall survival (OS) of most patients was not reached. Thus, the patients still need to be followed up. Thus, the evidence from current clinical practice is inadequate, and further clinical data are needed to confirm our results.

To our knowledge, this study is the first to report chemo-immunotherapy as a first-line treatment for advanced NSCLC with HER2 alterations. Overall, our study aimed to provide additional data regarding the treatment of NSCLC with HER2 mutation or amplification. The results suggest that chemo-immunotherapy may be a hopeful first-line treatment option for these patients. However, further clinical trials are required to expand treatment options for NSCLC patients with HER2 alterations.

## Data Availability Statement

The original contributions presented in the study are included in the article/supplementary material. Further inquiries can be directed to the corresponding authors.

## Ethics Statement

The studies involving human participants were reviewed and approved by The Committee on Medical Ethics of West China Hospital. The patients/participants provided their written informed consent to participate in this study. Written informed consent was obtained from the individual(s) for the publication of any potentially identifiable images or data included in this article.

## Author Contributions

SZ wrote the manuscript. SZ and XX collected the data. SZ and PT analyzed the data. KW and YL revised the manuscript. KW and WL designed the manuscript. KW and YL reviewed the manuscript. All authors contributed to the article and approved the submitted version.

## Funding

This work was supported by grants from National Natural Science Foundation of China (Grants 82070019 and 81870034) and Sichuan Science and Technology Program (No. 2020YFS0572).

## Conflict of Interest

The authors declare that the research was conducted in the absence of any commercial or financial relationships that could be construed as a potential conflict of interest.
